# Inflammasomes are important mediators of prostatic inflammation associated with BPH

**DOI:** 10.1186/s12950-015-0082-3

**Published:** 2015-05-17

**Authors:** Mahendra Kashyap, Subrata Pore, Zhou Wang, Jeffrey Gingrich, Naoki Yoshimura, Pradeep Tyagi

**Affiliations:** Department of Urology, University of Pittsburgh, Pittsburgh, USA

**Keywords:** Inflammasome, NLRP1, BPH, IL-18, Chemokines

## Abstract

**Background:**

There is mounting evidence to support the role of inflammation in benign prostate hyperplasia (BPH), and a recent study reported expression of inflammasome derived cytokine IL-18 in prostate biopsy of BPH patients. Here we examined the expression of inflammasome-derived cytokines and activation of nucleotide-binding oligomerization domain-like receptor with pyrin domain protein 1 (NLRP) 1 inflammasome in a rat model of prostatic inflammation relevant to BPH.

**Methods:**

Prostatic inflammation was experimentally induced in three-month-old male Sprague–Dawley rats by intraprostatic injection (50 μL) of either 5 % formalin or saline (sham) into the ventral lobes of prostate. 7 days later, prostate and bladder tissue was harvested for analysis of inflammasome by Western blot, immunohistochemistry and downstream cytokine production by Milliplex.

**Results:**

Expression of interleukins, CXC and CC chemokines were elevated 2-15 fold in formalin injected prostate relative to sham. Significant expression of NLRP1 inflammasome components and caspase-1 in prostate were associated with significant elevation of pro and cleaved forms of IL-1β (25.50 ± 1.16 vs 3.05 ± 0.65 pg/mg of protein) and IL-18 (1646.15 ± 182.61 vs 304.67 ± 103.95 pg/mg of protein). Relative to prostate tissue, the cytokine expression in bladder tissue was much lower and did not involve inflammasome activation.

**Conclusions:**

Significant upregulation of NLRP1, caspase-1 and downstream cytokines (IL-18 and IL-1β) suggests that a NLRP1 inflammasome is assembled and activated in prostate tissue of this rat model***.*** Recapitulation of findings from human BPH specimens suggests that the inflammasome may perpetuate the inflammatory state associated with BPH. Further clarification of these pathways may offer innovative therapeutic targets for BPH-related inflammation.

## Introduction

The prevalence of benign prostatic hyperplasia (BPH)/lower urinary tract symptoms (LUTS) in US population is expected to increase with an ageing population and an increased prevalence of metabolic diseases [1]. Although generally thought to be due to prostatic enlargement, BPH/LUTS is also known to be associated with intra-prostatic infiltration of inflammatory cells in majority of patients [2]. The presence of inflammatory infiltrates in the prostate biopsy predicted unfavorable outcomes in placebo-treated BPH patients in Medical Therapy Of Prostatic Symptoms MTOPS study [2].

Findings from large clinical studies suggest that LUTS, especially storage symptoms such as urgency and frequency, associated with BPH are not only a consequence of bladder outlet obstruction caused by prostatic enlargement, but there is also a contribution of prostatic inflammation. A large Olmsted county study on BPH patients found that daily use of a non-steroidal anti-inflammatory drug was inversely associated with onset of moderate/severe urinary symptoms [3]. Several other epidemiologic studies have also identified obesity as an important risk factor for BPH associated inflammation and most obesity related disorders are associated with excessive inflammasome activation [4]. While the expression of IL-1β and IL-18 were shown to be elevated in the prostate biopsy tissue obtained from BPH patients [5], none of the studies so far has examined whether this expression in the prostate is associated with inflammasome activation.

Inflammasomes are cytosolic oligomeric signaling platforms found in myeloid cells such as monocytes, macrophages and epithelial cells. Inflammasomes are thought to be responsible for initiating an inflammatory cascade in response to endogenous or exogenous stress signals [6]. In particular, NLRP (NLR family containing pyrin domain) inflammasomes are functional cytosolic homologs of membrane bound toll-like receptors [6] and crucial initiators of sterile inflammation in several metabolic disorders [7] and chronic inflammatory diseases [8, 9]. Interestingly, a recent report demonstrated that expression of mRNA coding for inflammasome components [10] was higher in prostate biopsy of BPH patients relative to the expression in prostate cancer patients. We have previously reported on a rat model of prostatic inflammation induced by intraprostatic formalin injection and its relevance for BPH studies [11]. In this study, we evaluated the expression of different chemokines including the activation of the inflammasome in prostate and bladder tissue following the induction of prostatic inflammation.

## Methods

All animal experiments were performed in accordance with institutional guidelines and with an approval from the University of Pittsburgh Institutional Animal Care and Use Committee (Protocol # 1011435). Male Sprague–Dawley rats weighing 250-320 g were anesthetized with isoflurane. Following an abdominal incision, formalin (5 % in saline) or saline (sham) was injected into each ventral lobe of the prostate (50 μl per lobe) to produce chemically induced prostatic inflammation. Ventral lobes of the prostate and bladder tissue were excised 7 days after injection.

### Histological analyses

A part of the prostate injected with either formalin or saline (*n* = 3 per group) was embedded in OCT Tissue-Tek compound (Sakura Finetek U.S.A, Torrance, CA), frozen on dry ice, and kept at -80 °C until use. Samples were serially sectioned at 8 μm thickness and stained with hematoxylin and eosin.

### Immuno-histochemical analysis

8 μm cryosections were washed in PBS and fixed in chilled acetone for 10 min at 4 °C. Sections were then incubated with PBS containing 0.4 % Triton X-100 (PBST) and 5 % normal donkey serum (Jackson Immunoresearch) for 30 min at room temperature. The primary antibodies specific to NALP1 (1:200, Abcam); and IL-18 (1:50, Santa Cruz Biotechnologies) were applied overnight to sections in PBST containing 5 % normal donkey serum at 4 °C. Sections were then washed 3 times with PBST containing 1.0 % BSA for 5 min at room temperature and then incubated for 2 h with secondary donkey anti-primary Alexa Fluor 488 or Alexa Flour 594 antibody (1: 200, Molecular Probes) at room temperature. Sections were washed again for three times at room temperature in PBST containing 1.0 % serum, and then mounted with medium containing 4’,6-diamidino-2-phenylindole DAPI (Floromount-G with DAPI, Fischer Scientific). Sections were visualized under Olympus BX51 microscope and the images were captured using MagnaFire 2.1 software.

### Measurement of chemokines

The prostate and bladder tissues of rats injected with formalin or saline (*n* = 6 per group) were homogenized using cold CelLytic™ MT Mammalian Tissue Lysis/Extraction Reagent (sigma) containing 2 mM sodium orthovanadate, 1 mM PMSF and protein cocktail inhibitor (1X, Sigma). The homogenate was centrifuged at 10,000 rpm for 10 min and the resulting supernatants were stored at -80 °C until assayed. 28 proteins including interleukins IL-1α, IL-1β, IL-2, IL-4, IL-5, IL-6, IL-10, IL-12p70, IL-13, IL-17A and IL-18; CXC chemokines (CXCL1, CXCL2, CXCL5 and CXCL10), CC chemokines (CCL2, CCL3, CCL5); Growth factors NGF, BDNF, VEGF and G-CSF, other inflammatory mediators such as eotaxin, leptin, IFN-γ and TNFα levels were determined on a Luminex 100 using a MILLIPLEX MAP Rat Cytokine/Chemokine Panel (Millipore, Billerica, MA). Levels of NGF and BDNF were determined using individual ELISA kits procured from Promega, USA. Protein estimation was done by BCA Protein Assay Kit (Pierce, Rockford, Illinois) to standardize the chemokine concentrations relative to tissue protein levels, which are expressed as pg/mg of total protein [12].

### Western blot analysis

Tissue was homogenized using CelLytic™ MT Mammalian Tissue Lysis/Extraction Reagent (Catalog no. C3228, Sigma, USA) in the presence of phenylmethylsulfonyl fluoride (1 mM), sodium orthovanadate (2 mM) and protein inhibitor cocktail (Catalog no. P8340-5ML, Sigma, USA). Protein estimation was done by Coomassie (Bradford) Protein Assay Kit (Thermo Scientific, USA). The lysates from the sham and formalin injected groups were separated on Tricine-SDS PAGE. Protein lysates (40 μg) were electrophoresed using 10 % Tricine-SDS Gel and then blotted onto immobilion-P membranes (Millipore) using wet transfer system. After blocking for 1 h at 37 °C, the membranes were incubated overnight at 4 °C with primary antibodies specific for NALP1 (1:500, Abcam), Caspase-1 (1; 500), IL1β (1:400) and β-Actin (1:1000, Santa Cruz Biotechnology), in blocking buffer (pH 7.5). The membranes were then re-incubated for 2 h at room temperature with secondary anti-primary immunoglobulin G (IgG)-conjugated with horseradish peroxidase (Santa Cruz Biotechnologies, USA). Subsequently, blots were developed using SuperSignal West Femto Maximum Sensitivity Substrate (catalog no. 34096, Thermo Scientific, USA) on Versa doc imaging system (Model 4000; BioRed, USA). Densitometry for measuring the band specific for each protein was performed using AlphaEase FC StandAlone V. 4.0.0 software. β-Actin was used as an internal control to normalize the data.

### Statistics

The unpaired Student’s *t*-test was used for comparing the values of two groups. All tests were two-sided, and p values < 0.05 were considered statistically significant. All statistical analyses were performed using Graphpad Prism IV.

## Results

### Chemokine expression in prostate & bladder

**Fig. 1:**
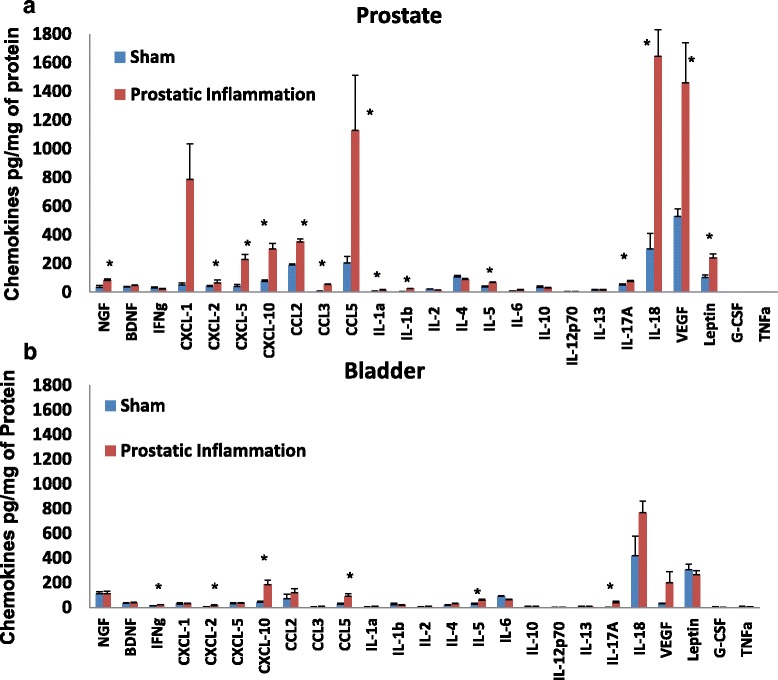
**a** Cytokine expression was elevated in prostate tissue harvested from prostatic inflammation group relative to sham. Relative to other cytokines, expression of IL-18 was quantitatively higher and also significantly relative to the expression of IL-18 in sham prostate. There was significant upregulation of IL-1α,IL-1β, IL-5, IL-17A, leptin, NGF, VEGF, CXC and CC chemokines (*p < 0.05, unpaired *t* test) in formalin injected prostate, while expression of BDNF, IFNγ, G-CSF, IL-2, IL-4, IL-10, IL-12p70, IL-13, TNFα remained unchanged between the groups. **b** Overall, expression of cytokines in bladder was lower relative to prostate (same y-axis scale). Bladder from sham group showed significantly lower expression of IFNγ, CXCL-2, CXCL-10, CCL5, IL-5 and IL-17A compared to the group injected with formalin (*p < 0.05, unpaired *t* test)

Expression of chemokines was elevated several fold in formalin injected prostate tissue relative to the expression of respective chemokines in saline injected prostate tissue (sham). All the chemokines belonging to CXC family were significantly upregulated in formalin injected prostate, with the maximum upregulation of 15 fold in mean levels of CXCL-1 followed by 5 fold upregulation of both CXCL-5 and CXCL-10 (Fig. 1a). Among CC chemokines, CCL3 showed a 9 fold upregulation, followed by a 5 fold upregulation for CCL5 and then 2-fold upregulation for CCL2 (*p < 0.05, unpaired *t* test). IL-18 expression was quantitatively higher relative to other cytokines/chemokines and the elevation of IL-8 in formalin injected prostate tissue was also significant relative to sham prostate tissue.

Among other interleukins, IL-1α, IL-1β, IL-5, and IL-17A were also significantly upregulated in formalin prostate, with the highest fold change noted for IL-1β. Among growth factors, prostatic inflammation induced a significant 3 fold upregulation of VEGF, NGF and two fold upregulation of leptin expression. The expression of BDNF, interferon-γ (IFNγ), G-CSF, IL-2, IL-4, IL-10, IL-12p70, IL-13, TNFα remained unchanged between the sham and formalin injected groups, whereas the expression of eotaxin was undetectable in prostate and bladder tissue of both groups.

Overall, bladder tissue expression of cytokines was lower relative to prostate of each group. Bladder tissue obtained from rat group given intraprostatic injection of formalin showed significantly higher expression of IFNγ, CXCL-2, CXCL-10, CCL5, IL-5 and IL-17A (*p < 0.05, unpaired *t* test). (Fig. 1b). The expression of all other proteins was not statistically different in the bladder tissue of the two groups.

### Western blot of inflammasome components

**Fig. 2:**
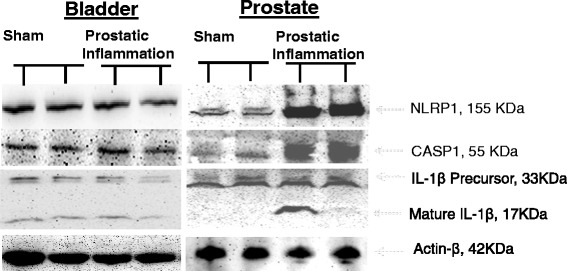
Western blot analysis for components of inflammasome in prostate and bladder tissue obtained from sham and prostatic inflammation group. Blots represent the activity of NLRP1, Caspase-1, parent and mature cleaved IL-1β in both tissues

Western blots of prostate tissue lysate showed significantly stronger density bands for NLRP1 (0.92 ± 0.02 vs 0.45 ± 0.01; *p < 0.05), Caspase-1 (0.97 ± 0.08 vs 0.42 ± 0.03;*p < 0.05) and mature IL-1β (0.40 ± 0.11 vs 0.08 ± 0.01;*p < 0.05) in rat group given intraprostatic injection of formalin relative to sham prostate (Fig. 2). In contrast, Western blot results for bladder showed absence of any change in expression of NLRP1 and other components following intraprostatic injection of formalin or saline.

### Immuno-histochemistry of inflammasome components

**Fig. 3:**
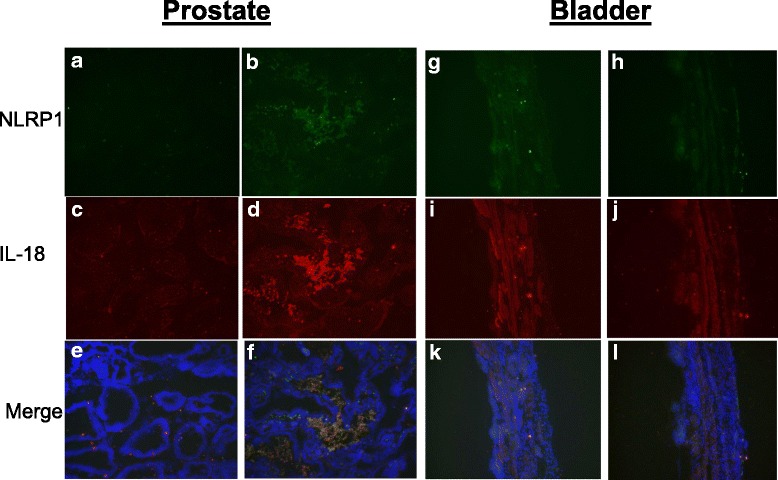
**a-f** Detection of NLRP1 inflammasomes (green) and derived cytokine IL-18 (red) in the prostate by immunohistochemistry in prostate of sham (panel **a, c** and **e**) and formalin injected group (panel **b, d** and **f**). Merged image in panel F shows the co-localized expression of NLRP1 with IL-8 against the DAPI blue background in formalin injected prostate and its marked absence in sham prostate. **g-l** Detection of NLRP1 inflammasomes (green) and derived cytokine IL-18 (red) by immunohistochemistry in bladder of sham (panel **g, i** and **k**) and formalin injected group (panel **h, j** and **l**). Merged image shows the constitutive expression of IL-18 against the DAPI blue background, but the absence of NLRP1 in bladder

Prostate from the formalin injected group showed higher green immunoreactivity for NLRP1 and red immunoreactivity for IL-18 protein relative to the sham group injected with the saline (Fig. 3a-f). The yellow signal seen in the merged image of panel F indicated the co-localized expression of NLRP1 with IL-18 against the blue DAPI background in the formalin injected prostate tissue. The merged image for the sham prostate tissue in panel E shows absence of the yellow signal, which confirms the lowered expression of NLRP1 and IL-18.

In contrast, there was no difference in the green immunoreactivity for NLRP1 and red immunoreactivity for IL-18 in the bladder obtained from sham (Fig. 3g, i and k) and formalin injected groups (Fig. 3h, j and l). The antibody for IL-18 performed better in immunohistochemistry than in Western blot (data not shown). Therefore, a role for inflammasome dependent cytokine expression is indicated in prostate tissue, but not in bladder tissue.

### Histology

**Fig. 4:**
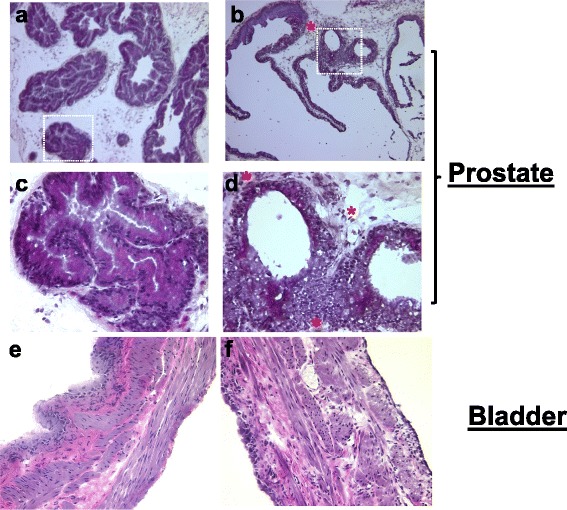
**a-d** Prostate tissue sections from sham group (panel **a** and **c**) showed regular shaped acini with an intact basement membrane. Prostatic inflammation in formalin injected group (panel **b** and **d**) caused epithelial pilling with budding into surrounding stroma at multiple foci. Expanded stromal area in formalin injected group showed inflammatory cells indicated by red colored * (panel **b** and **d**). Magnification in panel **a** and **b** is 10X and the region shown in white dotted line square region of panel a (sham) & b (prostatic inflammation) is magnified upto 4 fold and shown in panel c & d, respectively. **e-f** Bladder tissue sections obtained from sham group (panel **e**) showed no histological changes, whereas bladder from rat group with prostatic inflammation (panel **f**) showed slight edematous changes related to cytokine expression shown in Fig. 1b. Magnification is 20X in both panels

Prostate tissue obtained from the sham group showed regular shaped acini with an intact basement membrane (Fig. 4a and c). In contrast, formalin injected prostate tissue showed hyperplastic acini lined by tall columnar epithelium. Epithelial pilling with budding into surrounding expanded stromal areas was seen in multiple foci. Infiltration of inflammatory cells in the formalin injected prostate is indicated by the red color * (Fig. 4b and d). Bladder tissue from the sham group (Fig. 4e) showed histology similar to a normal, untreated rat. In contrast, bladder tissue from (Fig. 4f) the formalin injected group showed mild edematous changes, which were not accompanied by any marked infiltration of inflammatory cells.

## Discussion

Our previous study reported that clinical features, biochemical and histological changes in formalin induced non-infectious prostatic inflammation are highly similar to those reported for clinical BPH [11]. In the present study, we assessed the functional significance of inflammasome activation in the tissue specific expression of cytokines/chemokine in the prostate and bladder tissues of the same model. We observed that intraprostatic formalin injection leads to the assembly and activation of NLRP1 inflammasomes in prostate and production of pro-inflammatory cytokines, IL-1β and IL-18 following the auto-proteolytic maturation of cysteine protease, caspase-1. Meanwhile, the absence of inflammasome derived products in bladder suggests that chemokine expression in the bladder is induced by a stimulus, which is different from the prostate.

A role for NLRP1 inflammasome in prostate tissue has been previously demonstrated in another model of prostatic inflammation [13] induced by intraprostatic injection of carrageenan. Consideration of our findings, together with other similar reports, led us to propose that Nod-like receptor protein 1 (NALP1/NLRP1) inflammasomes mediate the prostatic inflammation in response to irritable stimuli such as formalin or carrageenan. Interestingly, detailed metabolic and molecular phenotyping in clinical studies have indicated that, inflammasome is a crucial link between BPH and metabolic disorders [10], since the inflammasome controls the energy expenditure and adipogenic gene expression [7] including that of adipocyte hormone leptin [14]. Enhanced leptin production noted in prostate after intraprostatic formalin injection mimics the likely endocrine influence from obesity in prostatic inflammation.

Visceral adiposity is correlated with circulating levels of pro-inflammatory cytokines, and adipose tissue is known to propagate inflammation locally and systemically, in part through chemokine mediated recruitment of macrophages. Expectedly, chemokines chemotactic for macrophages such as CCL2 were found elevated in prostatic fluids of BPH patients [15]. The upregulated expression of other CC chemokines, CCL2, CCL3 and CCL5 noted in formalin injected prostate is consistent with the prostatic infiltration of macrophages [16, 14]. Macrophages not only release CCL3 but, also serve as a site for inflammasome activation and are also known to positively regulate gene expression of CCL5 via NF-κB signaling cascades induced by IFN-γ [16, 14].

Inflamed regions in prostate biopsy of BPH patients are known to express upto 5-fold higher IFN-γ compared to the non-inflamed regions [17]. Here, we found that IFN-γ inducible chemokines [18] were upregulated to different extents in formalin-injected prostate tissue. CXC chemokines including CXCL1, CXCL2, CXCL5, are potent chemoattractants for neutrophils and can further drive the neutrophil dependent tissue injury [8] visualized on the histology of formalin injected prostate tissue. CXCL2 induces migration of hematopoietic stem cells and its expression is affected by FGF-2, which is implicated in BPH [19]. CXCL-10 is chemotactic for lymphocytes and is structurally and functionally different from CXCL-1, a rat homolog of human IL-8 [20].

The dysfunctional voiding reported in rats after intraprostatic formalin injection [21] is presumably linked to the predominant expression of IFN-γ and the IFN-γ inducible expression of chemokine, CXCL-10 in bladder. CXCL-10 is considered to be constitutively expressed in neurons and contribute to the excitability of primary afferent neurons through transactivation of transient receptor channels and nociceptor sensitization [22]. Clinical relevance of CXCL-10 in bladder function and urinary symptoms can be gleaned from the upregulated gene expression of CXCL-10 in bladder biopsy of ulcerative cystitis patients [23]. Interestingly, a previous report showed that systemic neutralization of CXCL-10 by monoclonal antibodies ameliorated the dysfunctional voiding following chemically induced cystitis [24]. Increased expression of CXCL-10 in bladder tissue of formalin injected rats accompanied a modest increase in the expression of CXCL-2, CCL5, IL-5 and IL-17A, which suggests a role for T-helper17 lymphocytes and epithelium in the observed bladder expression of chemokines [17]. Ubiquitous expression of IL-18 by most epithelial tissues [25] may explain the substantial IL-18 expression in bladder of both saline and formalin injected group.

Over expression of CC and CXC chemokines in prostate of formalin injected rats corroborates similar findings obtained in another rat model of BPH induced by chronic estradiol injection [26]. An earlier study mixed a colored dye with formalin during intraprostatic injection in order to check the spread of formalin outside of prostate tissue by the external spread of dye [21]. The study did not find any spread of the injected dye outside of the prostate, which precludes a possible direct irritation of bladder by formalin in this model. A severe inflammatory response typically seen with direct irritation of bladder by formalin [27] was also not seen in bladder histology images (Fig. 4f). Taking histology images of bladder together with NLRP1 blot and immunoreactivity indicates that inflammasome is not assembled in bladder tissue following intraprostatic injection of formalin. It is known that expression of NLRP3 is higher than expression of NLRP1 in bladder [6], but they both generate similar inflammasome derived products. Therefore, lack of any change in the expression of cleaved IL-1β or IL-18 in bladder tissue, lend further support to the absence of the inflammasome in the bladder of this model.

NLRP1 are known to respond to endogenous metabolic stress (ATP and fatty acids) [7], and to exogenous stress of microbial infection [6], but the precise stimulus triggering the activation of NLRP1 in human prostate remains to be investigated. Recent studies on tissue specimens of BPH patients have implicated a pivotal functional role for IL-18 in BPH [5]. Predominant expression of IL-18 in rat prostate of this model is supported by Western blot and immunoreactivity findings. The autocrine/paracrine actions of IL-1β secreted by the inflammasome are implicated by the overexpression of CC and CXC chemokines [28] in this model, as these mediators do not require inflammasome processing for secretion.

CC and CXC chemokines represent a large family of chemotactic peptides with a broad range of cellular targets generated by stromal and epithelial tissues of prostate and bladder. Leukocyte infiltration is the primary event in inflammation and expression of chemokines temporally precedes that infiltration, [29] which makes them well suited for characterizing disease phenotypes [30]. Here, we found organ-specific prominent molecular signatures of prostatic inflammation. Considering that NALP1/NLRP1 is a susceptibility gene involved in the devolvement of chronic inflammatory diseases [9], this model can be used to understand cellular triggers of inflammasome activation in BPH. Taken together, it is clear that the inflammasomes plays a role in prostatic inflammation associated with BPH and inflammasome targeted therapies could be an option for BPH management.

## Conclusions

Significant upregulation of NLRP1, caspase and downstream cytokines suggest that a NLRP1 inflammasome is assembled and activated in the prostate tissue of this rat model***.*** Recapitulation of findings from human BPH specimens suggests that the inflammasome may perpetuate the inflammatory state associated with BPH and further clarification of the pathways may offer innovative therapeutic targets in BPH.

## Abbreviations

NLRP1: Nucleotide-binding oligomerization domain-like receptor with pyrin domain protein 1
BPH: Benign prostatic hyperplasia
IL: Interleukins
MTOPS: Medical therapy of prostatic symptoms
LUTS: Lower urinary tract symptoms
NGF: Nerve growth factor
BDNF: Brain derived neurotrophic factor
VEGF: Vascular endothelial growth factor
G-CSF: Granulocyte stimulating factor
IFN-γ: Interferon-γ
